# Modular Energy-Efficient and Robust Paradigms for a Disaster-Recovery Process over Wireless Sensor Networks

**DOI:** 10.3390/s150716162

**Published:** 2015-07-06

**Authors:** Abdul Razaque, Khaled Elleithy

**Affiliations:** Department of Computer Science, University of Bridgeport, 126 Park Avenue, Bridgeport, CT 06604, USA; E-Mail: elleithy@bridgeport.edu

**Keywords:** wireless sensor networks, operational medium access control protocol, QoS, energy-efficiency, pheromone termite, disaster recovery

## Abstract

Robust paradigms are a necessity, particularly for emerging wireless sensor network (WSN) applications. The lack of robust and efficient paradigms causes a reduction in the provision of quality of service (QoS) and additional energy consumption. In this paper, we introduce modular energy-efficient and robust paradigms that involve two archetypes: (1) the operational medium access control (O-MAC) hybrid protocol and (2) the pheromone termite (PT) model. The O-MAC protocol controls overhearing and congestion and increases the throughput, reduces the latency and extends the network lifetime. O-MAC uses an optimized data frame format that reduces the channel access time and provides faster data delivery over the medium. Furthermore, O-MAC uses a novel randomization function that avoids channel collisions. The PT model provides robust routing for single and multiple links and includes two new significant features: (1) determining the packet generation rate to avoid congestion and (2) pheromone sensitivity to determine the link capacity prior to sending the packets on each link. The state-of-the-art research in this work is based on improving both the QoS and energy efficiency. To determine the strength of O-MAC with the PT model; we have generated and simulated a disaster recovery scenario using a network simulator (ns-3.10) that monitors the activities of disaster recovery staff; hospital staff and disaster victims brought into the hospital. Moreover; the proposed paradigm can be used for general purpose applications. Finally; the QoS metrics of the O-MAC and PT paradigms are evaluated and compared with other known hybrid protocols involving the MAC and routing features. The simulation results indicate that O-MAC with PT produced better outcomes.

## 1. Introduction

Wireless sensor networks (WSNs) are considered to be appealing event-based systems that achieve both densely deployed sensor nodes and the perception of a certain physical sensation. The key objective is to consistently sense the event features while utilizing minimal energy resources, processing and storage capabilities [1,2]. To achieve this objective, a substantial amount of research has been conducted to develop the cooperative networking protocols needed to accomplish the necessary communication while achieving maximum energy efficiency. Most of the communication protocols for WSNs are individually designed and developed for different layers, *i.e.*, the physical, medium access control, network and transport layers. These communication protocols have achieved significant improvements in terms of the different parameters correlated with each of the individual layers. However, these protocols experience several challenges when combined for multiple layers; these challenges include additional energy consumption, QoS, mobility, scalability, uniformity, data redundancy, congestion, lack of robustness, reliability and insufficient coverage. Furthermore, the limited battery life and extreme operating conditions can cause node failure [3], which wastes additional energy [4]. Considering these challenges faced by WSNs, a multi-layered network design, *i.e.*, a cross-layer paradigm, is considered to be the most promising substitute and has attracted increasing interest from the research community in recent years. There are a few cross-layer protocols for maintaining high standards of communication, particularly coverage, but the issues of high energy consumption and improving the QoS have not been properly resolved [4]. With the proper placement of a mobile infrastructure, a wide area can be covered compared with an infrastructure-based network deploying the same number of sensors [5]. However, the key services’ energy efficiency and the QoS provision are hurdles for robust WSNs. Detailed studies in [6,7,8] show that the cross-layered approaches could result in substantial savings in terms of energy efficiency. As a result, several cross-layered approaches have been designed and implemented [9,10,11,12,13]. A review of these approaches reveals that they either provide logical and analytical results without any proper communication protocol design or accomplish cross-layer support only within a restricted scope, e.g., MAC and routing layers. There is still much to be gained by reconsidering the features and functionalities of the protocol layers in an integrated manner to introduce communication paradigms for efficient and reliable communication while saving energy and improving the QoS provisioning in WSNs. To handle these issues, this paper introduces modular energy-efficient and robust paradigms that improve the energy efficiency and QoS provisioning. These paradigms involve the O-MAC protocol with the PT model to build a hybrid approach by incorporating the medium access control with the network. The O-MAC protocol involves the features of time division multiple access (TDMA) and carrier sense multiple access (CSMA). The O-MAC maintains the trade-off between energy efficiency and time management by introducing an optimized data frame format model. The optimized data frame format (ODFF) replaces the existing IEEE 802.15.4 data frame format model by modifying the existing features and incorporating new features, e.g., a reduction in the size of the existing preamble, which helps to save energy and improve the QoS parameters. The new features also include the anycast addressing methodology and automatic buffering because the anycast methodology helps to select the particular nodes in the 1-hop neighborhood to avoid the overhearing problem and thus reduces the additional energy consumed in the 1-hop neighborhood; furthermore, automatic buffering helps to reduce the data storage wait time. The TDMA part of O-MAC is also improved because the nodes are not required to synchronize with all of the 1-hop neighborhood nodes but rather only with the particular nodes that must be used for forwarding the data to the next hop of the network. In addition, ODFF includes the security portion to avoid the attacks encountered at the transceiver of the radio. To handle the collisions in accessing the slots at the channel, a novel randomization algorithm is introduced for energy efficiency and QoS provisioning. For example, sensor node “A” wants to access a slot number 5, and if slot number 5 is already occupied by node “B”, then this algorithm helps to redirect node “A” to another available empty slot to avoid the potential collision on the network. Extending the network lifetime, a head node is dynamically selected in each region. Furthermore, the PT model introduces significant features e.g., the pheromone sensitivity, which helps in predicting the amount of the pheromone and packet generation rate and in determining the traffic-forwarding link capacity to send the packets according to the capacity of the link. As a result, the PT model provides the shortest path from the source to the destination without overlap. In addition, it is also capable of load balancing and fault tolerance in the case of route failure and possible congestion. 

Therefore, O-MAC with PT maintains the state of the art in terms of energy efficiency and also develops the tradeoff between energy efficiency and time management. In the simulation platform, the state-of-the-art hybrid and individual layered configurations have been implemented to obtain a complete performance evaluation. The simulation experiments and analytical performance and evaluation demonstrate that O-MAC with PT substantially improved the communication performance and outperformed other competing hybrid protocols in terms of energy efficiency and QoS parameters. These results highlight the benefits of the novel modular energy-efficient and robust paradigm (O-MAC with PT) for deploying general scenarios as well as particular scenarios in WSNs. 

The remainder of this paper is organized as follows: in Section 2, we present the system model for the proposed work. In Section 3, the O-MAC protocol design goals and architecture are described. In Section 4, the pheromone termite model is presented. In Section 5, the simulation setup and evaluation of the results is discussed. Finally, Section 6 concludes the paper. 

## 2. System Model

The disaster recovery process facilitates an effective recovery response to disaster-congested states, territories, tribes, and local dominions. The robust disaster recovery process provides a flexible archetype that empowers the disaster response staff to function in a cohesive and collective manner. It also emphasizes the importance of reestablishing, regenerating and rejuvenating the social, health, natural, economic, and environmental aspects of the community and shaping a more vigorous community. Thus, this type of recovery process requires a state-of-the-art wireless sensing paradigm that helps eliminate the various restrictions related to traditional wireless networks. The WSNs are deployed to support several application areas. We introduce the flat topology network based on our proposed paradigms for monitoring the disaster recovery situation. The network is divided into three regions, and each region is handled and controlled by a dynamic operational node (DON). The DON acts as a powerful cluster head node in each region and has added capabilities, e.g., high energy efficiency, substantial memory allocation resources and additional data-forwarding capacity. 

**Figure 1:**
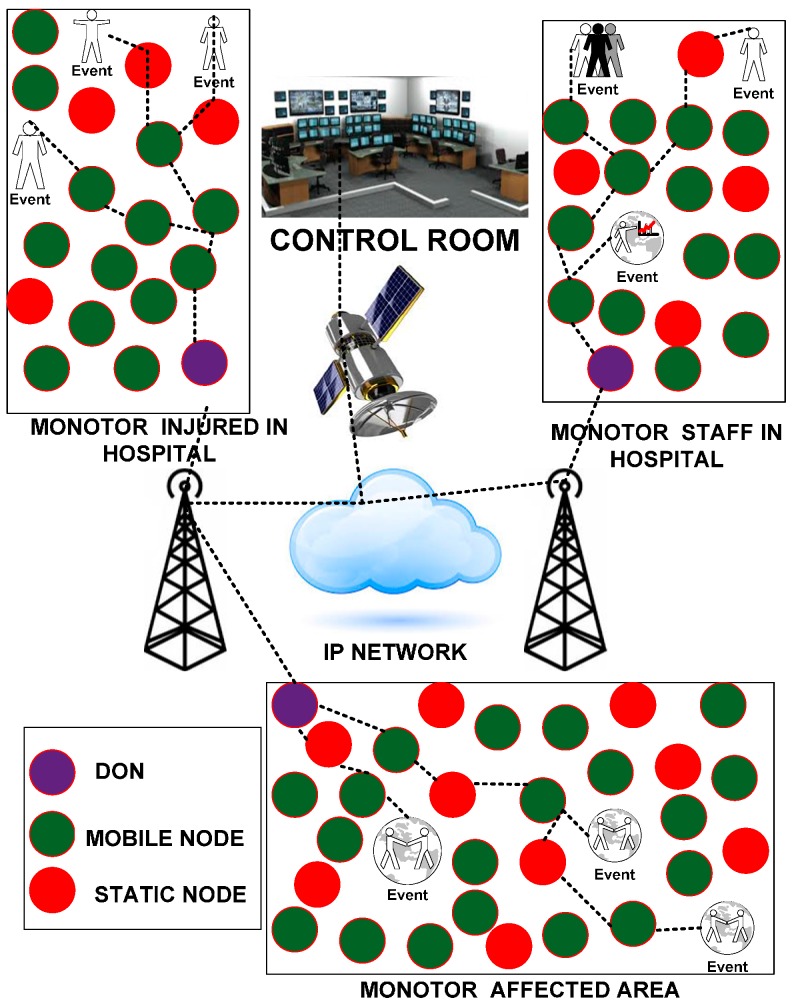
Deployment of a WSN for monitoring the disaster recovery process.

Each network region monitors the different events of the disaster recovery operation for the collection of the data depicted in Figure 1. In each region, there are three events to be monitored. Region “A” monitors the activities of the rescue team members and victims of the disaster. In region “A”, 90% of the nodes are mobile sensors with human-sensual capabilities attached to the body of rescue team members for collecting and processing the sensory information of victims and coordinating with mobile sensors attached to other staff (e.g., ambulances, fire brigades, firefighters). We assume that the other 10% are static sensor nodes that are already deployed for the protection of each region. Region B monitors the performance and activities of doctors, nurses and other staff in the disaster situation so that their roles and effectiveness in responding to the disaster situation can be analyzed. In region “B”, 50% of the nodes were assumed to be static and deployed in different parts of the hospital building, and the remaining 50% were mobile sensor nodes attached to the body of doctors, nurses and other staff. Region “C” collects updated information regarding victims who were brought into the hospital for treatment. In region “C”, 20% of the nodes are mobile sensor nodes that are attached to the body of wounded people because static sensor nodes could increase the anxiety level of those injured persons, which would be challenging for the staff to manage.

This situation could be highly annoying when the injured are shifted from one unit to another because static sensor nodes would need to be removed and reattached. Keeping with the vision of faster and reliable communication, the mobile sensor nodes are the best replacement for the jumble of wires and wounded people’s concerns. Triage protocols are already available for handling such emergency-based situations [14]. However, all of these existing protocols lack support for mobility. In addition, 80% of the sensor nodes are static and deployed inside the rooms to monitor and report the current status of the wounded. In handling this disaster situation, the static and mobility-aware nodes use the semi-synchronization approach to synchronize with each other. Semi-synchronization is a novel approach that helps to select the particular nodes in the 1-hop neighborhood for communication, and this approach consumes only the energy of those nodes that are randomly selected based on their available resources (e.g., energy, bandwidth, and shortest distance), as explained in [15]. The semi-synchronization approach is deployed for the nodes in regions that are following the interior communication process explained in the next section. The semi-synchronization is managed by deploying the anycast methodology.

The anycast methodology helps forward the sensor data to the next hop nodes. The nodes in each region forward the collected data of each event to the DON. Thus, efficient bandwidth utilization and bidirectional end-to-end reliability are the focus to maintain the smooth transfer of data from a region’s nodes to the DON because they are mandatory in WSNs [16,17,18]. End-to-end reliability is acknowledged when the data from each event are reported to the DON. The DON of each region forwards the collected data from the nodes in regions applying exterior communication to the base station using the IP network. The DON uses the purely scheduled based approach of time division multiple access (TDMA). We assume that the transmission range of the mobile and static sensor nodes is the same as that of the high-power sensor nodes. Therefore, each sensor node is enabled to communicate within a 1-hop neighborhood. Each sensor node collects the data from its region only. There are two types of communication: one is between sensor nodes and the DON, and the other is from DON to DON in the case of a greater distance of the DON from the base station. Therefore, TDMA is combined with frequency division and multiple access (FDMA) to enable concurrent communication among the sensor nodes [19]. The frequency channels are recycled throughout the simulation process in the entire WSN so as to minimize the required number of frequency channels. Consequently, the sensor nodes and DON can communicate self-reliantly without snooping each other. 

We have considered the industrial, scientific and medical (ISM) bandwidth for the WSN. The entire bandwidth is 2.4 GHz and is initially distributed into two portions with a ratio of 1:6. The larger 2.0 GHz portion is assigned to the nodes of the region, and the smaller 0.4 GHz portion is allocated to the DONs that use the FDMA technique for communication. The bandwidth is distributed among the sensor nodes such that each neighboring sensor node uses a different frequency band. The PT routing paradigm is used for data routing. PT involves two important features: the packet generation rate, the pheromone sensitivity, which helps determine the predicted amount of pheromone, and each link’s capacity. The packet generation rate helps determine how many packets could be generated. As such, this feature provides advanced information regarding the flow of traffic that helps avoid congestion over the network. In addition, pheromone sensitivity predicts the amount of traffic on each link and helps in forwarding the data to each link according to the capacity, which maintains load balancing. If this system model is deployed in a realistic environment, it could provide both operational stability and a swift recovery for critical scenarios. It would also guarantee that the disaster recovery process could be accurately monitored and communicated to all staff, clearly recognizing all of the essential roles and their responsibilities using WSNs.

## 3. O-MAC Protocol Design Goals and Architecture

For several applications, hybrid approaches are preferred over individual technology approaches in terms of energy efficiency, scalability and QoS provisioning. To improve the energy efficiency, scalability and QoS, O-MAC with the PT routing paradigm is designed and implemented. The O-MAC protocol leverages the features of both CSMA and TDMA. O-MAC uses a novel ODFF model for CSMA as a replacement for the IEEE 02.15.4 data frame format, whereas TDMA deploys the semi-synchronized scheduling methodology rather than traditional scheduling approach. The semi-synchronous approach helps to obtain faster access to the medium [20]. In our case, we deploy a semi-synchronized approach to handle the additional energy consumption that is consumed in the overhearing problem. The ODFF incorporates new features, such as a reduction in the size of the short preamble, introduction of the anycast methodology, automatic buffering, and insertion of a security portion. In addition, O-MAC involves three modules: interior communication, exterior communication, and dynamicity of the operational node. These three modules aim to improve the QoS provision and energy efficiency. As a result, the network lifetime is extended. The interior communication module uses the anycast methodology, semi-synchronized features, automatic buffering and randomization. The randomization feature is used to handle the congestion in the network when two nodes attempt to access the same slot in the channel. We achieve the following goals using O-MAC:

- Reducing the time and energy consumption for channel access,
- Reducing the time and energy consumption for loss (retry) packets,
- Reducing the time and energy consumption for acknowledgement,
- Reducing the time and energy consumption for data forwarding,
- Handling the overhearing and avoiding congestion in the network,
- Improving the QoS provisioning (e.g., throughput, latency, bandwidth utilization), and
- Extending the network lifetime.

### 3.1. Interior Communication

This process involves the communication of a region’s nodes using the flat network topology. In this process, the sender uses the semi-synchronization methodology to synchronize the particular nodes in the 1-hop neighborhood. The semi-synchronization methodology does not require synchronization with all of the nodes but rather selects particular nodes within the 1-hop neighborhood based on the shortest distance, energy and buffer capacity of each node. Our scheme addresses the pair of nodes needed to forward the data in each neighborhood: the principal and backup nodes; however, each sender stores the information of all nodes if the principal and backup nodes run out of energy or cease communicating. Thus, in the situation of handoff or loss of a node (e.g., failure of node, depleted node energy), other nodes are chosen as the next principal and backup nodes. The selection process for the principal and backup nodes is explained in Algorithm 1. 

**Algorithm 1** Selection process for the principal and backup nodes
Initialization of resources (Δ*D_s_*: Shortest Distance, Δ*E*: Energy, Δ*B_c_*: Buffer Capacity)Initialization of Nodes ($P_{n}$: Principal Node & $B_{n}$: Backup Node, $N_{n}:$ Sensor node, &: Candidate Node)Initialization of network ($R_{A}$: Region of Network)**While** *‘*$S_{n}$*’* selects $P_{n}$ & $B_{n}$ nodes in $R_{A}$**If** $S_{n}\to C_{n}$ = $\left\{N_{n}\;/\;N_{n}\in \;R_{A}\;;\;\forall \;\;N_{n}\leq 1\leq R_{A}\;\right\}\;\equiv$*△D_s_***Set** $△D_{s}\;\exists \ll \;C_{n}$**end if****If** $S_{n}\to C_{n}=\left\{N_{n}\;/\;N_{n}\in \;R_{A}\;;\;\forall \;\;N_{n}\leq 1\leq R_{A}\;\right\}$ ≡*△E* + *△B_c_*$△E\;+\;△Bc\;\exists \ll \;C_{n}$**end if****Compute** $C_{n}=△E\;+\;△Bc+\;△Ds$**set** $P_{n}||B_{n}=C_{n}\;$**end while**

In line 4, the sender node attempts to determine the principal and backup nodes in the interior region of the network. In line 5, the checking process for identifying the candidate node is performed. In line 6, the initial candidate node is selected based on the shortest distance from the sender node. Subsequently, in lines 8 and 9, a candidate node is selected based on the greatest energy and buffer capacity. In line 11, the weight of the candidate node is computed based on the distance, energy and buffer capacity. Finally, the node with the maximum weight is selected as either the principal node or the backup node in line 12.

The chosen principal node takes on the responsibility of forwarding the data to the next 1-hop neighbor node. Furthermore, in the case of a loss or handoff of a principal node, a backup node replaces the principal node for forwarding the data. Subsequently, another backup node is selected within the 1-hop neighborhood. When either the principal or backup node hand off or begin to run out of energy, each of the nodes (principal and backup) must incorporate a flag signal in the last packet sent that indicates the current situation of the node. This feature also helps the node to adjust for mobility. To forward the data to the next hop, the anycast addressing methodology is introduced to manage either the principal or backup node. The anycast addressing methodology is not a feature of the IEEE 802.15.4 data format packet, but is introduced in the ODFF model, which helps manage particular nodes in the 1-hop neighborhood to forward the data to the next hop. The interior communication process also involves the randomization function to avoid congestion when the node attempts to access a slot in the channel. For example, when node A wants to access slot 5, if slot 5 is already occupied by node D, then the randomization function redirects node A to another available empty slot to avoid any possible collision in the channel. When node acquires access to a channel, it uses the short preamble prior to sending the original data. The impact of the reduced sized preamble introduced in the ODFF is a tradeoff between the time and energy efficiency in the network. We also prove the tradeoff impact of O-MAC with other MAC protocols that use the preamble-based approach, as explained and analytically validated in the next section. The interior communication involves the following phases:

- The ODFF model.
- The preamble tradeoff validation model.

#### 3.1.1. Optimized Data Frame Format Model

**Figure 2:**
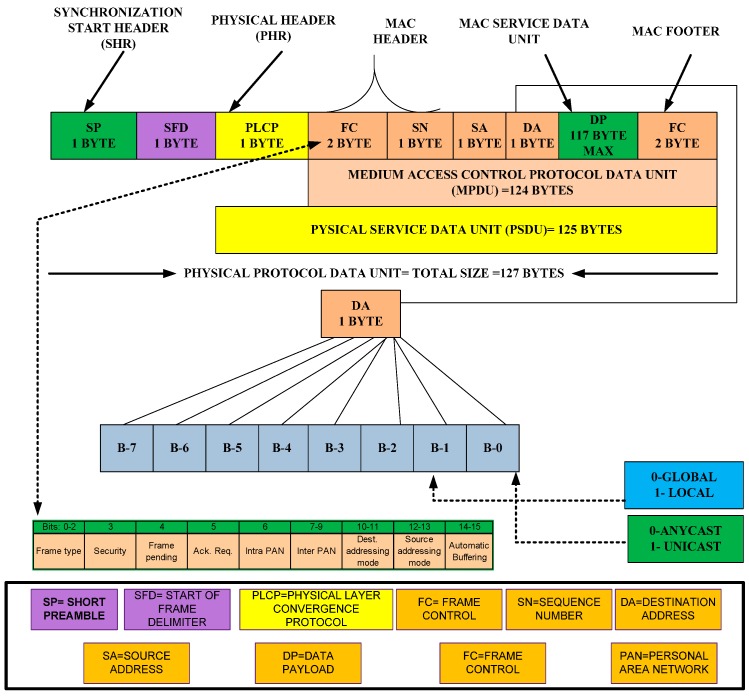
Optimized data frame format.

The goal of the ODFF is to enable sensor nodes to maintain QoS provisioning. The ODFF is preferred over the IEEE 804.15.4 standard and modifies existing features and includes new features depicted in Figure 2. The architecture of the ODFF is compatible with a robust and reliable wireless link. The ODFF can individually detect every radio in a WSN as well as the process and layout of communications between the radios. It also works beyond the physical and MAC layers, providing the cross-layered support to connect with the routing protocol.

The ODFF reduces the channel access time, data transfer time, preamble transfer time, acknowledgement time, and retry transmission time because the performance of small devices—especially sensor nodes—depends on the reduced amount of time. Hence, the timing overhead can affect the lifetime of the node. In addition, the ODFF can be used with low-duty-cycle MAC protocols due to the reduced preamble size, addressing fields, addition of automatic buffering and employment of the anycast message addressing methodology. These modifications also enable the ODFF to transfer more data compared with the IEEE 802.15.4 standard. Based on the modifications and inclusion of new features, we focus on the following parameters to determine the impact.

■ Channel access time.
■ Data frame transfer time.
■ Transmission timing for acknowledgement.
■ Retry transmission.
■ Possible transfer data rate in the ideal and worst-case scenarios.

##### Channel Access Time

In this section, the ODFF improves the CSMA portion of IEEE 802.15.4 and determines the time consumed in accessing the channel. As in IEEE 802.15.4, a sensor node in the ODFF must wait for a random back-off period within the range of {0, 2^γ^−1}. The node can wait for the channel up to 2^γ^−1 in the case of congestion, and then, it begins to access the channel again. Here, we set a value of 3 for “γ”. Thus, the channel access time can be obtained as:

$$\text{∆φ}+c=\left(2^{\text{γ}}-1\right)\times {\left(U_{bt}\right)}^{2}+\text{ω}\times \;\text{β}$$

Hence:

(1) $$\begin{array}{c} C_{at}=\text{∆φ}+c\\ C_{at}=\text{∆φ}+c=\left(2^{\text{γ}}-1\right)\times {\left(U_{bt}\right)}^{2}+\text{ω}\times \;\text{β}\; \end{array}$$

We use a maximum wait time of ω = 8 to send the data over the channel after a collision to determine whether there is any further possibility of a collision. Each symbol takes 16 µs. 

##### Data Frame Transfer Time

The ODFF reduced the preamble size from 4 bytes to 1 byte because the 4-byte preamble size creates overhead and reduces the throughput. Based on the standard transfer rate of the modem, we have calculated the data frame transfer time as:

(2) $$T_{f}=\frac{\left(P_{max\;}+P_{l}+\;F_{dil}+F_{l}\right)\times \text{ω}\;}{\text{∆}R}$$

##### Transmission Timing for Acknowledgement

**Figure 3:**
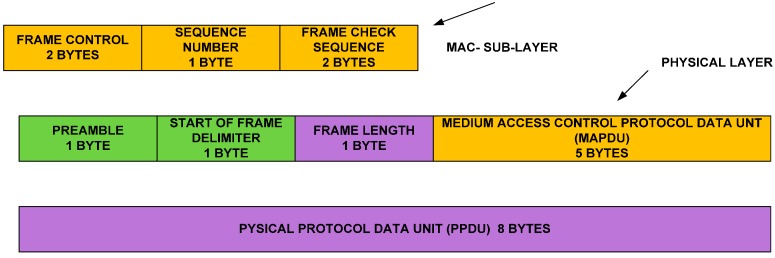
Acknowledgement data frame format.

We have already indicated that our objective is to reduce the preamble size. Therefore, the ODFF uses an 8-byte acknowledgement frame that can reduce the substantial amount of time in the acknowledgement frame, as shown in Figure 3. Based on the standard data rate of the modem, we can calculate the acknowledgement transmission time “$T_{ack}$” as:

(3) $$T_{ack}=\frac{\left(M_{pdu}+P_{l}+\;F_{dil}+F_{l}\right)\times \text{ω}\;}{\text{∆}R}$$

##### Retry Transmission

The node can wait for a particular amount of time for the MAC acknowledgement prior to attempting a retry transmission. We have set a wait time of 48 symbols. Each symbol takes 16 µs. If the number of symbols increases, the transmitting node could stay for a longer time in an ideal mode. Based on our experiments, we have determined that the ideal wait time for MAC could be 30, 36, 42, 48 or 54 symbols. We chose 48 symbols, but a smaller number could be chosen instead. If the network consists of multi-hops, then a smaller number is not an ideal choice for a large-scale wireless sensor network. Therefore, a 48-symbol wait time works perfectly on single-hop as well as multi-hop WSNs. Thus, the retry transmission time can be calculated as follows:

(4) $$T_{retry\;}=T_{s\text{∆}}\times \;\text{β}\;\;$$

##### Possible Transfer Data Rate in the Ideal and Worst-Case Scenarios

The possible data rate can be determined using an ideal scenario and a worst-case scenario. In an ideal situation, the ODFF-based approach finds the free channel for sending the data on a non-beacon-enabled network. The ODFF also improves the data payload and reduces the acknowledgement time. In the worst-case scenario, it should be assumed that 25% of the data packets require a retry transmission. Thus, the possible total time in the ideal and worst-case scenarios can be calculated as follows:

(5) $$T_{ti}=C_{at}+T_{f}+T_{turn}+\;T_{ack}$$

Equation (5) shows the total time consumed for transmitting the data frame in an ideal scenario.

(6) $$I_{data}=\frac{\left(D_{p}\;\times \;\text{ω}\;\right)}{T_{ti}}$$

Equation (6) shows possible data rate in an ideal situation using the ODFF approach.

(7) $$T_{tw}=C_{at}+T_{f}+T_{retry\;}+C_{at}\;+\;T_{f}+T_{turn}+\;T_{ack\;\;\;}$$

Equation (7) shows the total time consumed for transmitting the data frame in the worst-case scenario.

We have already calculated the total time in ideal scenario “$T_{ti}$” consumed by the 75% of the data frame packets that do not require a retry transmission; 25% of the data frame packets are assumed to experience a retry transmission. Thus, the data rate in the worst-case scenario can be calculated as follows:

(8) $$\begin{array}{c} T_{time}=\left(T_{ti}\times 75\%\right)\;\;+\left(T_{tw}\times 25\%\right)\\ W_{data}=\frac{\left(D_{p}\;\times \text{ ω}\;\right)}{T_{time}}\;\; \end{array}$$

When a specific amount of data is transmitted on network using ODFF approach, the total time can be calculated as follows:

(9) $$T_{td}=\frac{S_{d}}{D_{p}}\;\times T_{time}\;\;$$

Based on this mathematical formulation, we have validated the important parameters of our proposed ODFF and the existing IEEE802.15.4 data frame formats. The statistical data are shown in Table 1.

**Table 1:** Comparison of BN-MAC and IEEE 802.15.4 based on the data frame model.

Name of the Parameter	Analytical Calculation for the Optimized Data Frame Format	Analytical Calculation for IEEE 802.15.4
Chanel access time	Cat = (2^3^ − 1) × 16×16 + 8×16 = 1.92 ms	2.368 ms
Data frame transfer time	$T_{f}=\frac{\left(127+1+1+1\right)\times 8\;}{250\;\times \;10^{3}}=4.16\text{ ms}$	4.256 ms
Transmission timing for acknowledgement	$T_{ack}=\frac{\left(5+1+1+1\right)\times 8\;}{250\;\times \;10^{3}}=0.256\text{ ms}$	0.352 ms
Turnaround time	0.192 ms	0.192 ms
Retry transmission time	$T_{retry\;}=48\times 0.016=0.768\text{ ms }$	0.864 ms
Total access channel, frame transfer and acknowledgement and turnaround time in an ideal scenario	$T_{ti}=1.92+4.16+0.192+\;0.256=6.528\text{ ms}$	7.168 ms
Total access channel, frame transfer and acknowledgement and turnaround time in the worst-case scenario	$T_{tw}=1.92+4.16+0.768+1.92+4.16+0.192+\;0.256=13.376\text{ ms}$	14.656 ms
Time to send 1 MB of data	$T_{td}=\frac{2^{20}}{117}\;\times 8.24\;=70\;$ s	

Based on the obtained statistical results, the ODFF is considerably better than the existing IEEE 802.15.4 protocol in terms of each parameter; therefore, the ODFF can extend the network lifetime and improve the QoS. The details of the metrics used in the ODFF are provided in Table 2.

#### 3.1.2. Preamble Tradeoff Validation Model

The O-MAC uses the ODFF model, which is a tradeoff between the energy consumption and time. The ODFF comes with a short preamble that shows an improvement in the QoS and energy efficiency compared with existing lower-power listening (LPL) protocols. The LPL protocols use a long-sized preamble prior to sending the data on the channel. The long-sized preamble takes additional time to reach to other nodes. As a result, additional time and energy are consumed at non-targeted receivers. The LPL protocols also introduce an additional latency at each hop. The long preamble-enabled protocols have a serious problem because all of the nodes over a channel must wait until the long preamble is delivered to the targeted node. This scheme consumes additional energy at both the sender and receiver. For example, in X-MAC [21], the destination address is incorporated within each preamble, which not only increases the size but consumes additional energy and requires additional time to arrive at another node. Despite the address of a particular node having been merged into the packet, each node checks all of the preamble packets on the network. As a result, all of the nodes along the way consume time and additional energy because the sensor nodes are not intelligent. If a preamble packet is discarded by a non-intended node, then there is no possibility for the preamble to be delivered to the anticipated node. If the node is the anticipated recipient, it remains awake for the subsequent data packets. 

**Table 2:** Description of the notations used in the ODFF.

Notation	Description/Definition
$\left(2^{\text{γ}}-1\right)$	Total number of slot waits
β	Required time for sending each symbol
c	Congestion
$C_{at}$	Channel access time
$D_{p}$	Data payload
$F_{dil}$	Frame delimiter used for giving the signal of the preamble
$F_{l}$	Frame length
$M_{pdu}$	Medium access control protocol’s data unit length
$P_{l}$	Packet length
$P_{max}$	Maximum number of packets
$T_{retry}$	Retry transmission
$T_{f}$	Data frame transfer time
$T_{ti}$	Total time in the ideal scenario
$T_{time}$	Total time spent in the worst-case scenario
$T_{s\text{∆}}$	Total symbol wait time for the acknowledgement
$T_{tw}$	Total time in the worst-case scenario
$T_{turn}$	Processing time
ω	Number of symbols
∆R	Standard transfer rate
∆φ	Initial back-off time used to give space to other nodes to access the channel

**Figure 4:**
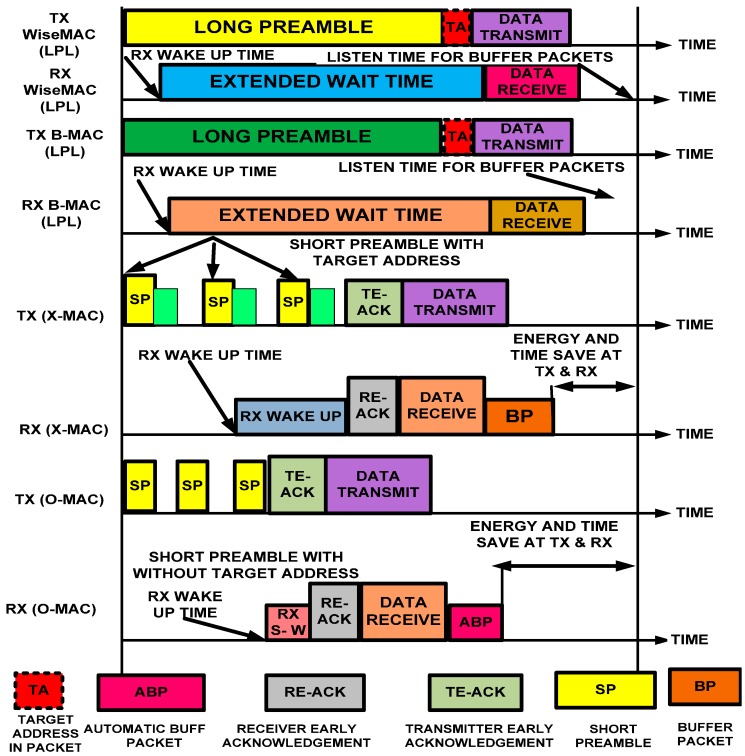
Timeline comparison of low-duty-cycle hybrid MAC protocols.

Furthermore, X-MAC is purely based on an asynchronous approach, and it does not follow the neighboring nodes’ schedules. As a result, the nodes consume additional energy while waiting on the channel for the next traffic to be called, which is an idle listening problem. The reduced size of the preamble in O-MAC confirms and validates the tradeoff between time and energy. We demonstrate the processes of B-MAC [22] and WiseMAC [23] (long preamble-enabled MAC protocols), short preamble (X-MAC) and O-MAC in Figure 4. As noted above, O-MAC uses an ODFF that not only has support for a short preamble but also provides an automatic packet buffering process. Thus, it reduces the wake up time and consumes less energy. 

We analytically proved in the previous section that use of a short preamble consumes less time for several parameters; here, we validate the energy consumed by a short preamble and by the sending and receiving of data over the channel. 

The energy consumed for the short preamble can be obtained as:

(10) $$E_{sp}=\text{∆p}+\;{E_{syn}}^{2}\times \;C_{drift}\;$$

The node that transmits its clock to the one-hop neighbor during interior-region-communication is called the source node, and the node that receives the clock in the one-hop neighborhood is called the particular node (either the principal or backup node). The synchronized nodes send a short preamble prior to sending data without using the target address because a short preamble is sent to particular nodes in the one-hop neighborhood, which reduces the energy consumption. 

Let us assume that the energies consumed in one work cycle by the source and the particular nodes are “β” and “δ”, respectively. The average short preamble reception time could be reduced because the particular node wakes up based on the stored schedule. Thus, the source node and particular node consume an amount of energy that can be calculated as follows:

(11) $$\text{β}=\overset{n}{\underset{i=0}{\sum }}S_{j}\frac{\left(\text{∆φ}.\mu \times \text{∆}v^{2}\;\right)\times \left(\;{E_{syn}}^{2}\times \;C_{drift}\right)+\left(E_{ct\;\;\;+}\text{∆p}\right)\;}{{\text{∆}}_{t}}\;$$

Equation (11) yields the energy consumed by the source node.

(12) $$\text{δ}=\overset{n}{\underset{i=0}{\sum }}S_{j}\frac{\left(\text{∆φ}.\mu \times \text{∆}v^{2}\;\right)\times \left(\;{E_{syn}}^{2}\times \;C_{drift}\right)+\left(E_{ct\;\;\;+}\text{∆p}\right)\;}{{\text{∆}}_{t}}\;+\frac{\left(\;{E_{syn}}^{2}\times \;C_{drift}\right)+\left(E_{ct\;\;\;+}\text{∆p}\right)\;}{{\text{∆}}_{t}}\;\left(12\right)$$

Equation (12) yields the energy consumed by the particular node that is available at the one-hop destination. Table 3 provides the factors used in the preamble tradeoff validation model. 

**Table 3:** Notations and system definitions.

Notations	System Definitions
C	Collision on the channel
C_drift_	Clock drift
$E_{ct}$	Energy consumed during the channel access time
$E_{sp}$	Energy consumed by the short preamble
${E_{syn}}^{2}$	Energy consumed by synchronization at both the transmitter and receiver sides
I	Starting number of the short preamble
N	Ending number of the short preamble
$S_{j}$	Short preamble
$\overset{\cdot }{\text{S}}$	Symbol sent over the channel to determine the availability of the channel
${\left(U_{bt}\right)}^{2}$	Unit backup time to avoid deadlock at the channel
∆φ	Size of the short preamble
∆p	Average energy consumed by carrier sensing
∆φ	Initial backup time
${\text{∆}}_{t}$	Time consumed for sending the short preamble
$\text{∆}v^{2}$	Short preamble speed
$\overset{\cdot }{\text{t}}$	Check time
β	Energy consumed by the source node
δ	Energy consumed by the particular node (principal or backup node)
μ	Nature of the location

To validate the analytical model, we have substituted the equations and compared the results for X-MAC, IEEE 802.15.4, B-MAC and WiseMAC. We are interested in determining the energy consumed for the metrics, such as the channel access, the short preamble, and data forwarding, which are calculated and given in Table 4. 

**Table 4:** Time and energy consumption of MAC protocols.

Parameters	Preamble-Enabled MAC Protocols				
	WiseMAC	B-MAC	X-MAC	O-MAC	IEEE802.15.4
Energy consumed to access the channel	0.886 × 10^−6^ Joules	0.896 × 10^−6^ Joules	0.839 × 10^−6^ Joules	0.619 × 10^−6^ Joules	0.936 × 10^−6^ Joules
Energy consumed to send the short preamble	1.832 × 10^−6^ Joules	1.842 × 10^−6^ Joules	1.659 × 10^−6^ Joules	1.152 × 10^−6^ Joules	1.805 × 10^−6^ Joules
Energy consumed to forward 5 MB of data	0.964 × 10^−1^ Joules	0.962 × 10^−1^ Joules	0.928 × 10^−1^ Joules	0.821 × 10^−1^ Joules	0.976 × 10^−1^ Joules

**Figure 5:**
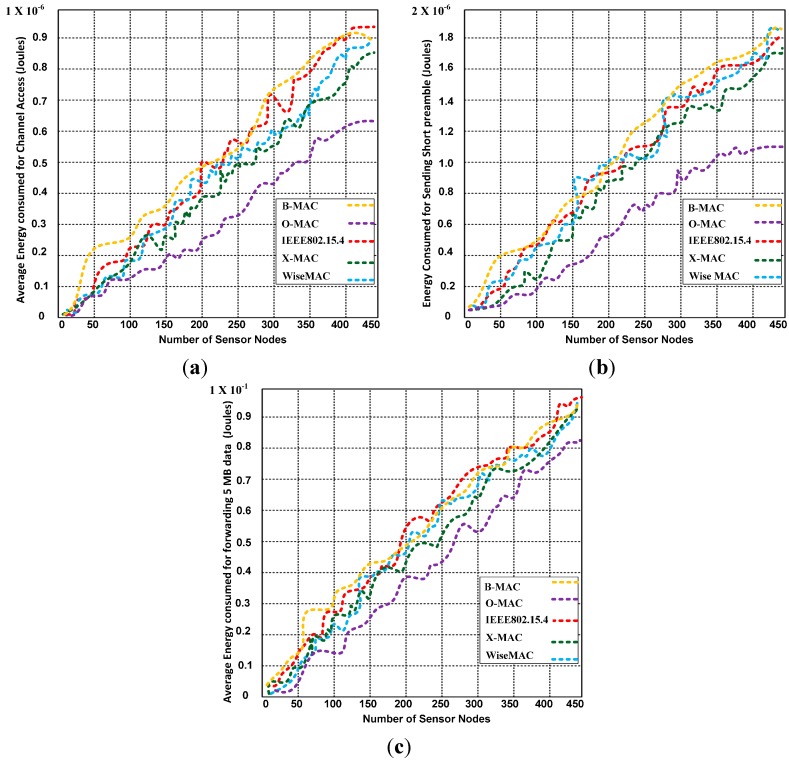
(**a**) Average energy consumed to access the channel; (**b**) Average energy consumed to forward the short preamble and (**c**) Average energy consumed to forward 5 MB of data (Joules).

Furthermore, to confirm the analytical results shown in Table 4, we have programmed and simulated the preamble-enabled protocols using the network simulator-3 (NS3). The simulation results were identical to those obtained through the analytical calculation. Based on the simulation results, we depict the energy consumed for the channel access, short preamble and data forwarding in Figure 5.

### 3.2. Exterior Communication

This section describes how to fix the schedules within the regions and exterior to the regions (base station or adjacent region). Each region of the WSN is managed and controlled by the DON, which collects the data packets from interior nodes and forwards the data packets to the exterior region. The DON of each region uses a schedule-based mechanism to communicate either interior or exterior nodes. When a DON wants to share its schedule with interior nodes, it first broadcasts three “hello” messages to warn the interior nodes to be ready for receiving the dynamic operational node indication signal (DONIS). The DONIS contains the current time, next collection time, and next delivery time. The current time consists of the current activities of the DON. The next collection time gives the complete schedule to the interior nodes and also collects the data from them. The next delivery time indicates when the DON must forward the collected data packets from the interior nodes to the base station or adjacent DON. If the interior region is far from the base station, the DON must synchronize with another DON to forward the collected data packets from the interior nodes to the base station. All of the interior nodes are active only during the DONIS to receive the schedule from the DON. Once the DON announces the schedule for the interior nodes, all of the nodes must follow the announced schedule. The working process for exterior communication is described in Algorithm 2.

**Algorithm 2** Exterior Communication Process**Input** (*D_s_*: Dynamic operational node indication signal, $A_{ck}$: Acknowledgement & $\hat{\text{H}}$: Hello messages)**Output** ($EX_{t}$: Exterior traffic, & $EN_{t}$: Exterior traffic)**Parameters** ($C_{t}$: Current time, $CA_{t}$: Next collection time, & $D_{t}$: Next delivery time)**Initialization of network** (BS: Base station, : Adjacent region, $D_{on}$: Dynamic operational node, $S_{ns}$: Sensor nodes of region $T_{n}$: Total nodes, & $R_{A}$: Region of Network)**if** $D_{on}$ forwards $\hat{\text{H}}$ into $R_{A}$ then;**Set** $S_{ns}$ response with $A_{ck}$**end if****if** $D_{on}$ gets $A_{ck}$ then;**Set** $D_{on}$ broadcasts *D_s_***Set** *D_s_ =*$C_{t}+\;CA_{t}+D_{t}\;$**end if****if** $S_{ns}$ receive *D_s_* then;$S_{ns}$ forwards $EN_{t}$ to $D_{on}$**for (**$S_{ns}=\;0;\;S_{ns}\leq \;T_{n};\;S_{ns}+1)$**Store** $EN_{t}=\;EN_{t}+\;S_{ns}$**Set** $EX_{t}\;\approx \;\;EN_{t}$**end for****Set** $D_{on}$ forwards $EX_{t}$ to $BS\;||\;A_{R}$**end if**

In line 5, the DON forwards 3 “Hello” messages to the region’s nodes to ensure that they are ready to receive the DONIS. In line 6, the region’s nodes respond to the DON with an acknowledgement. In lines 8–9, the DON receives the acknowledgements and forwards the DONIS to the region’s nodes. Line 10 shows the contents of DONIS. In lines 12 and 13, once the sensor nodes receive the DONIS, they respond by forwarding the data of the sensed events to the DON for the end node. In lines 14 and 15, the data of the exterior’s nodes are stored in the buffer of the DON during the allocated scheduled time. In lines 16 and 18, the DON converts the collected interior traffic into exterior traffic for forwarding to either an adjacent region or the base station.

### 3.3. Dynamic Operational Node

The DON is selected based on the residual energy, data-forwarding capacity and buffer allocation resources. We assign a weight to each factor. The node with the highest weight is selected as the DON. Once the DON completes 10 cycles, a base station broadcasts the message inside the network for the selection of a new DON. After receiving the message from the base station, the nodes with substantial data-forwarding capacity are declared as the candidate DONs. Subsequently, each sensor node determines its residual energy and buffer allocation resources.

The advantage of this approach is that it provides a sufficient number of options to each node to be declared the DON based on the set criteria. We describe the residual energy process of each node in each region. Let us assume that single-hop communication is used among the sensor nodes to detect events and transmit the information. Each node forwards data at some specified distance within the region and located within the N * N area of the WSN. We determine the residual energy of two types of nodes: the DON and the non-dynamic operational nodes (NDONs), which can be expressed as described below. 

A DON requires three types of messages for communication, with their corresponding consumed energies: “${\text{ E}}_{\text{sch}}$” for scheduling, “${\text{E}}_{\text{adv}}\;$” for the advertisement and “${\text{E}}_{\text{dat}}$” for sending the data. Thus, the energy consumed by a DON for scheduling can be computed as follows:

(13) $$E_{sch}=\frac{n\left(\text{∆}C_{p}\times R_{e}\right)+n\left(A_{e}\times \text{∆}C_{p}\right)}{2M_{e}}+r^{2}\;\left(N-1\right)\;$$

Equation (13) provides the energy consumed in scheduling the nodes.

(14) $$E_{adv}=\frac{\left(\text{∆}C_{p}\times R_{e}\right)+\left(A_{e}\times \text{∆}C_{p}\right)}{2M_{e}}\;$$

Equation (14) provides the energy consumed in advertising the message for the interior and exterior communication.

(15) $$E_{dat}=\frac{{\left\{n\left(\text{∆}D_{p}\times R_{e}\right)+n\left(A_{e}\times \text{∆}D_{p}\right)\right\}}^{2}}{2M_{e}}+\;r^{2}\;\left(N-1\right)$$

Equation (15) provides the energy consumed in receiving the data from the region’s nodes (interior communication) and in forwarding the data to either the base station or adjacent DON of another region. 

By combining Equations (13)–(15), we can determine the total energy consumed by the DON for the entire communication process. Finally, the energy consumed by the DON will be subtracted from the initial energy, which yields the residual energy of the DON, as given in Equation (16):

(16) $$DE_{res}=\left[E_{ini}-\;\left\{\frac{n\left(\text{∆}C_{p}\times R_{e}\right)+n\left(A_{e}\times \text{∆}C_{p}\right)}{2M_{e}}+r^{2}\;\left(N-1\right)\right\}+\;\left\{\frac{\left(\text{∆}C_{p}\times R_{e}\right)+\left(A_{e}\times \text{∆}C_{p}\right)}{2M_{e}}\right\}+\left\{\frac{{\left\{n\left(\text{∆}D_{p}\times R_{e}\right)+n\left(A_{e}\times \text{∆}D_{p}\right)\right\}}^{2}}{2M_{e}}+\;r^{2}\;\left(N-1\right)\right\}\right]\;$$

Similarly, we can determine the total energy consumed by the NDONs, but the NDONs do not use an advertisement message in our case. The NDONs only use “$E_{sch}$” and “$E_{dat}$” messages. Thus, the consumed energy of the NDONs is obtained from Equation (17):

(17) $$NDE_{res}=\left[E_{ini}-\;\left\{\frac{n\times \left(\text{∆}C_{p}\times R_{e}\right)+n\times \left(A_{e}\times \text{∆}C_{p}\right)}{2M_{e}}+r^{2}\;\left(N-1\right)\right\}+\left\{\frac{{\left\{n\times \left(\text{∆}D_{p}\times R_{e}\right)+n\times \left(A_{e}\times \text{∆}D_{p}\right)\right\}}^{2}}{2M_{e}}+\;r^{2}\;\left(N-1\right)\right\}\right]\;\;\;$$

Based on Equations (16) and (17) and the data-forwarding capacity and buffer allocation, a new DON is selected. The notation is described in Table 5.

**Table 5:** Notation and its description.

Notation	Description
$A_{e}$	Energy consumed in amplifying the signal
$E_{adv}$	Energy consumed in advertisement
$E_{dat}$	Energy consumed in sending the data
$E_{ini}$	Initial energy of the border node or non-border nodes
$E_{res}$	Residual energy of the node after performing several cycles
$E_{sch}$	Energy consumed in synchronization
$2{\text{ M}}_{e}$	Mean energy consumed by the radio and in amplifying the signal
n	Number of messages
N	Number of nodes
$r^{2}$	Number of hops
$R_{e}$	Energy consumed by the radio signal
$\text{∆}D_{p}$	Data packets
$\text{∆}C_{p}$	Control packets
$DE_{res}$	Residual energy of a DON
$NDE_{res}$	Residual energy of NDONs

## 4. Pheromone Termite Analytical Routing Model

Pheromones are the chemicals used for communication. Pheromone communication is conceivably the most ancient means of communication in the animal realm. The animals transmit the chemical messages that include information regarding food location, territory, threats, and companions. The chemical messages are private, potent in darkness, and long-term, and they may function over long distances. Here, we specifically consider pheromones as used by termites. Termites use pheromones for communication purposes. When termites search for food, they use a pheromone to mark the track from the food to their shell. The termites deposit the pheromone from a gland on the bottom of their stomachs. When termites are back at the nest, they recruit other termites to follow the trail of the pheromone back to the food. Based on the idea of termites’ use of pheromones, we introduce the pheromone termite model with new features added for routing purposes. Our pheromone termite model adds two additional new features into the existing working process of the pheromone termite model: the packet generation rate and pheromone sensitivity. The packet generation rate helps avoid congestion, and the pheromone sensitivity helps determine the link capacity prior to sending the packets on each link. These two additional features provide a tradeoff between fault tolerance and QoS provisioning. 

Here, we use the PT model with O-MAC to obtain cross-layering support. The model with two additional new features connects the MAC sub-layer with the network layer to find the optimal path to transmit the data packets through one-hop neighbor nodes. We achieve the following goals using the PT model.

- Low latency,
- Higher throughput,
- No-overlapping,
- Collision/congestion handling,
- Fault-tolerance, and
- Use of the shortest paths.

The working process of the PT model starts once a carrier is accessed to forward the data packets using the optimal path. We use an analytical mathematical computation to determine the effectiveness of the PT model with the two additional features.

Let us assume that “$C_{R}$” is the communication range and that the distance between the two nodes is “r” in meters. The force is inversely proportional to the distance [24]. Therefore, we can apply the free space propagation model to measure “$C_{R}$”, as shown in Equation (18):

(18) $$C_{R}=\frac{T_{X\;}R_{X}P_{t}{\text{λ}}^{2}}{\left(4{\text{π}}^{2}\right)r^{2}N_{l}}\;$$

where “$R_{X}$” and “$T_{X}$” are the energy gains of the receiver and transmitter, respectively, in watts, $N_{l}\;\left(N_{l}\;\geq 1\right)$ is the network loss, and “λ” is the wavelength in meters/second. It is typical to set $R_{X}=T_{X\;}=1$ and $N_{l}=1$.

The distance between the two sensors, “r”, is periodically calculated after a time interval to generate new entries in the table. This process removes the old entries and calculates “r”, which is used to update the trajectory pheromone in the sensor nodes. The trajectory pheromone is the group of termites extending from the source to the destination (l,s). We deploy the features of the trail pheromone and ant control algorithm [25]:

(19) $$\underset{\;\;l,s}{P^{\left(N\right)}}=\{\underset{\;\;l,s}{P^{\left(N\right)}}\times e^{-\left(rc-rsn{)}^{\beta }\right)+}\;P^{a\;\;};\;\;l=\;h^{p}$$

where $\underset{\;\;l,s}{P^{\left(N\right)}}$ is the number of pheromones (total number of packets transmitted over the network). from the source sensor node “s” that are forwarded onto the link to the one-hop neighbor “*l*” for node “n”, h^p^ is the previously determined hop of the packet, “P^a^” is the amount of pheromone that each packet carries, “r_c_” is the current distance to the neighbor node “n” at link “l”, “e” is the distance from the same neighbor node at link “l” to the destination node where the last packet was delivered, and “β” is the packet generation rate. The calculated trail pheromone (initial control message sent by a termite either for discovery of the route or for informing the next termite to follow the chosen route) is used to determine the forwarding power (power capacity for sending the packets) of each neighbor node, which can be calculated as:

(20) $$\underset{\;\;q,r}{P^{\left(N\right)}}=\;\frac{{\left(\underset{\;\;q,r}{P^{\left(N\right)}}+\text{Ƈ}\right)}^{P_{s}}}{\sum_{u=1}^{K}{\left(\underset{\;\;u,r}{P^{\left(N\right)}}+\text{Ƈ}\right)}^{P_{s}}}\;$$

where P^(N)^_q,r_ is the power strength of each neighbor node “u” that forwards the packet destination “r” to node “n”, “K” is the total number of neighbor nodes, “Ƈ” is the pheromone threshold, which is a constant, and “$P_{s}$” is the level of pheromone sensitivity. In a WSN, two nodes work as routers to establish the communication link to send the data packets. The pheromone threshold and pheromone sensitivity (the detection power—emitted signal—that the termite uses to communicate with other termites) can also be used to find the second-best alternate path to forward the packets to the desired destination.

We determine an average predicted amount of pheromone “Pψ” on different links of the WSN. Let us assume that “X” is the source node and “Y” is the destination node that uses multiple links ($Link_{1},\;Link_{2},\;Link_{3},\;.\;.\;.,Link_{n}$) to send the pheromone (carrying or transferring the substance as a signal to other termites for communication). Each link has attributes that are characterized by a non-negative random operation λo(r) with a mean value of Ф_o_(r). Each packet carries a fixed amount of pheromone “P^a^”. Let us assume that each node generates the pheromone at a constant rate “β”. Suppose that two nodes, X and Y, are located at two locations separated by a distance “r” and are uniformly distributed over the WSN. The sensor node distance distribution is applied using a Rayleigh distribution [26]. If the transmission power of a sensor node is less than the WSN area, then the separation distance is divided into a range from 0 to r, and the distribution can be described as a probability density function, as follows [27]:

(21) $$V(r)=\frac{{R_{e}}^{-R\text{β}}/\left(2R^{2}\right)}{R^{2}}\;\;$$

where V(r) is the node separation distribution, which can be used to compute the predicted pheromone generation $P\left(Re^{-R\text{β}}\right)\;$at a node separation distance “r” that corresponds to the number of arrival packets. This probability density function is used to determine the density of the WSN.

Let us assume that “Z” is used to describe the rate of pheromone generation $Z=\left(Re^{-R\text{β}}\right)$ at a node separation distance “r” that corresponds to the packet arrival rate. 

Thus, we can obtain the node separation distribution V(r), the rate of pheromone generation “Z”, and the separation distance “r”. $V(r)=\frac{\frac{R_{e}-R\text{β}}{2R^{2}}}{R^{2}}\;\;for\;0\;\leq r\;\leq R\;and\;Z=\left({R_{e}}^{-R\text{β}}\right)$; then:

$$r=\;\frac{-\log\;z}{\text{β}}$$

We use the generated pheromone “Z” as the input and apply the node separation distribution V(r) to determine the total generated predicted pheromone as follows:

$$V\left(Z\right)=\frac{{R_{e}}^{-R\text{β}}}{2R^{4}}\;\frac{\left(\log_{Z}\right)}{\text{β}}\times \;\left|\frac{-1}{Z\text{β}}\right|$$

Enumerating the order of equalities results in:

(22) $$V\left(Z\right)=\frac{{R_{e}}^{-R\text{β}}}{2R^{4}}\times \;\frac{1}{Z}\;\left(-logz\right)\;\;\;{R_{e}}^{-r^{2}}\;\leq Z\;\leq 1\;\;$$

Thus, the predicted generated pheromone can be calculated as follows:

(23) $$\begin{array}{c} P\left(R_{e}-r^{2}\right)=P\left(Z\right)=\int_{{R_{e}}^{-R\beta }\;\;}^{0}\frac{2}{R^{2}\beta^{2}}\;\;*\;\frac{1}{Z}\times Z\;\left(–logz\right)rz\\ P\left(R_{e}{{-}^{R\beta }}^{-r^{2}}\right)=\;\frac{2}{R^{2}\beta^{2}}\;\;\int_{R_{e}{-}^{R\beta }{-}^{R\beta }\;\;}^{0}-logz\;(\;rz)\\ P\left(Re^{-r^{2}}\right)=\frac{2}{R^{2}\beta^{2}}\;[-\left(zlogz-z\right)\;{]}^{1\;\;\;}\;R_{e}{-}^{R\beta }\\ P\left({R_{e}}^{-r2}\right)=\frac{2}{R^{2}\beta^{2}}\;\;\left[1-\;R_{e}{-}^{R\beta }\;\left(R\beta +1\right)\right]\;\;\; \end{array}$$

The predicted pheromone generation rate is used to compute an average predicted amount of pheromone on single and multiple links using the pheromone update generation function. However, the analytical model of pheromone generation (Packet generation rate) must be validated. Let us assume that “P” is the population at a distance “h” and that “P_i_” is the initial population (Number of termites at the beginning of activity (sensor nodes) taking part in performing the activities). Therefore, $\frac{rp}{h}\;=\;\text{β}P_{i}$.

Substituting the value of p_i_ yields Equation (24):

where $P_{I}=rh$

(24) $$\begin{array}{c} \frac{rp}{h}\;=\;\text{β}\times rh;\;rp\;=\;\text{β}\times rh^{2};\;P\;=\text{ β}\times h^{2}logp=\;-\text{β}\times h+ώ\\ p=\text{ω}\times \;{R_{e}}^{-\text{β}*h} \end{array}$$

The pheromone update function can also be written as:

(25) $$p=\text{ω}\times \;{R_{e}}^{-\text{β}*h}\;\;=\;\;\text{ω}\times \;{R_{e}}^{-h}\times \text{β}\;\;\;$$

This function is used to calculate an average predicted amount of pheromone on single and multiple links. Based on the following assumptions, an average predicted amount of pheromone on a single link can be obtained using the pheromone update function for several packets “n” consecutive times.

For a number of delivered packets, let us assume the following:

- The number of delivered packets for a distance “r” is a Poisson distribution (The number of dropped packets in the network) with an average wavelength $\;\lambda =\frac{v}{f}$ packets/meter. These packets consume resources in the network.
- The average amount of received pheromone is “Pψ”.
- The initial amount of pheromone on a single link is “P_i_”.

Thus, the pheromone update equation is used “n” consecutive times to determine the number of delivered packets on a single link:

(26) $$p\left(n\right)={R_{e}}^{-}\;\left(\overset{n}{\underset{x=0}{\sum }}r_{x}\right)\text{β}+p\text{ψ}\times \;\overset{n}{\underset{y=1}{\sum }}R_{e}-\left(\overset{n}{\underset{x=y}{\sum }}r_{x}\right)\text{β}\;\;$$

The predicted amount of pheromone PP(n) for “n” delivered packets can be calculated as follows:

(27) $$\begin{array}{c} PP\left(n\right)=p_{i}\times {\left(\;P\left({R_{e}}^{-r^{2}}\right)\right)}^{n}\;+\;p\text{ψ}\;\left(\frac{(P{\left(R_{e}{{-}^{r}}^{2}\right)}^{n}}{P\left(R_{e}{{-}^{r}}^{2}\;\right)}\right)\\ PP\left(n\right)=\;p_{i}\;{\text{σ}}^{n}+\;p\text{ψ}*\left(\frac{1-{\text{σ}}^{n}}{1-{\text{σ}}^{n}}\;\right)\;\; \end{array}$$

Thus, the predicted amount of pheromone for “n” arrived packets “PP(r)” on a single link for a node separation distance “r” is expressed as a Poisson distribution:

(28) $$f\left(z\right)=\;\frac{\lambda^{z}\times {R_{e}}^{-\lambda }}{Z!}$$

where “λ” is the average number of successfully received packets, “Z” is the number of successful attempts in which we are interested, and “R_e_” is the base of the logarithmic function. We map and apply the Poisson distribution in our problem; the details are as follows:

(29) $$\begin{array}{c} PP\left(r\right)=\sum_{i=0}^{\infty }[poisson\;\left(\lambda r,n\right)\left[PP\left(n\right)\right]\\ PP\left(r\right)=\sum_{i=0}^{\infty }[{R_{e}}^{-\lambda r}\frac{\left(\lambda r\right)}{i!}\;]\times \;[p_{i}\;{\text{σ}}^{n}+\;p\text{ψ}\times \left(\frac{1-{\text{σ}}^{n}}{1-\text{σ}}\;\;\right)]\\ PP\left(r\right)=\frac{p\text{ψ}}{1-{\text{σ}}^{n}}\;+\;{R_{e}}^{-\text{λ}r}\frac{\left(\text{λβ}r\right)}{\lambda +\text{β}}\;\;\left(p_{i}-\;\;\frac{p\text{ψ}}{1-\text{σ}}\;\right)\;\;\; \end{array}$$

An average pheromone performance for a longer time can be obtained as follows:

$$\underset{r\to \infty }{\lim}\;PP\;\left(r\right)=\;\frac{p\text{ψ}}{1-\text{σ}};\;PP\left(r\right)=\;\frac{p\text{ψ}\;\left(\text{λ}+\text{β}\right)}{\text{β}};\;and\;\text{λ}=\frac{v}{f};$$

Thus:

(30) $$PP\left(r\right)=\;\frac{p\text{ψ}\;\left(\frac{v}{f}+\text{β}\right)}{\text{β}}\;\;$$

If we use only a single link to send the packets, then PP(r) provides the predicted amount of pheromone on the single link. We can determine the predicted amount of pheromone on multiple links in a similar manner. On multiple links, the value of the constant pheromone threshold “Ƈ” is set to 0, and the pheromone sensitivity level “P_s_” is 1. 

Let P_0_, P_1_, P_2_, …, P_n_ be the multiple links that forward the data over the WSN with a packet generation of $P\left(Re^{-R\text{β}}\right)$. Thus, the average pheromone for the multiple links can be calculated as follows:

(31) $${P_{0}}_{Y}^{X}=\;{P_{0}}_{Y}^{X}\times \left[\frac{2}{R^{2}{\text{β}}^{2}}\;\left[1-{R_{e}}^{-\text{β}R}\;\left(1+\text{β}R\right)\right]\right]+\;\left[\;\frac{{\left({P_{0}}_{X}^{Y}\;+\text{Ƈ }\right)}^{P_{s}}}{{\left({P_{0}}_{X}^{Y}\;+\text{Ƈ }\right)}^{P_{s}}+{\left({P_{1}}_{X}^{Y}\;+\text{Ƈ }\right)}^{P_{s}}+{\left({P_{2}}_{X}^{Y}\;+\text{Ƈ }\right)}^{P_{s}}+,\;.\;.\;.,{\left({P_{n}}_{X}^{Y}\;+\text{Ƈ }\right)}^{P_{s}}}\;\right]\times p\text{ψ }$$

A termite maintains the pheromone table to store information about each neighbor node. Similarly, each node maintains a routing table to preserve the amount of pheromone for each neighbor link. The node possesses a different pheromone trail and table in the form of a matrix with listed destination nodes, including side nodes and neighboring nodes, across the top. Rows represent the destination nodes, and columns represent the neighbor nodes. An entry made in a pheromone is represented by $P_{k,r}$, where “k” is the neighbor index and “r” is the destination index, as explained in [28]. The value stored in pheromone Table 6 helps in calculating the probability of each node.

**Table 6:** Pheromone routing table for node N.

Neighbor/Destination Node	V	W	X	Y	A
B	P_V,B_	P_W,B_	P_X,B_	P_Y,B_	P_A,B_
C	P_V,C_	P_W,C_	P_X,C_	P_Y,C_	P_A,C_
F	P_V,F_	P_W,F_	P_X,F_	P_Y,F_	P_A,F_
D	P_V,D_	P_W,D_	P_X,D_	P_Y,D_	P_A,D_
V	P_V,V_	P_W,V_	P_X,V_	P_Y,V_	P_A,V_
W	P_V,W_	P_W,W_	P_X,W_	P_Y,W_	P_A,W_
X	P_V,X_	P_W,X_	P_X,X_	P_Y,X_	P_A,X_
Y	P_V,Y_	P_W,Y_	P_X,Y_	P_Y,Y_	P_A,Y_
Z	P_V,Z_	P_W,Z_	P_W,Z_	P_Y,Z_	P_A,Z_

**Figure 6:**
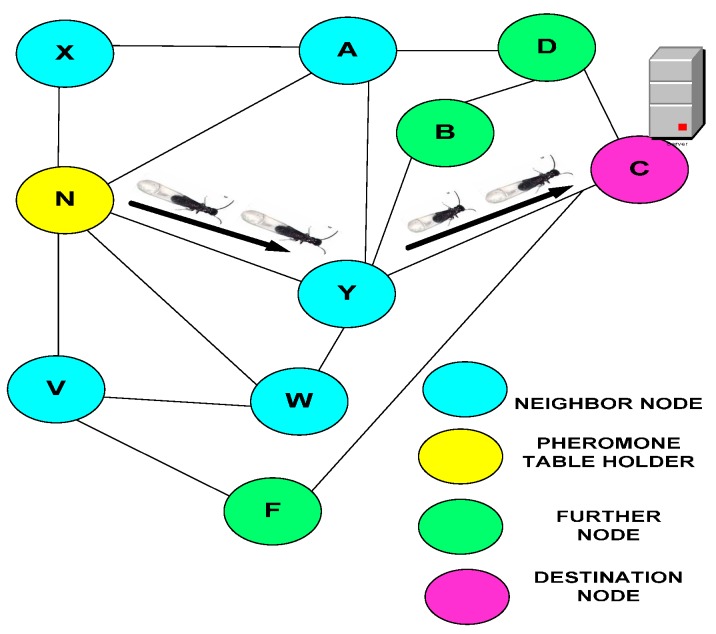
Routing process of the pheromone termite model.

Figure 6 depicts the search process of the termite. When a packet is received at node “N” from the previous hop “$P_{hop}$”, that node could be a source node. Thus, the source, pheromone decay and pheromone are added to link $\underset{NP_{hop}}{\to }$. Therefore, node “N” consists of A, V, W, X, and Y neighbor nodes. The shortest link to reach the desired destination node is $\underset{NYC}{\to }$. The possible links to forward the data to destination C are $\underset{NXADC}{\to }$, $\underset{NADC}{\to }$, $\underset{NVFC}{\to }$ and $\underset{NNWYC}{\to }$, but $\underset{NYC}{\to }$ is the shortest link to reach the destination. Thus, the entire path-following process helps find the shortest path from the source to the destination with minimal overhead.

## 5. Simulation Setup and Result Analysis

We have generated and simulated a disaster recovery scenario that covers indoor and outdoor activities using the ns-3.10 network simulator on the Ubuntu 14.04 operating system. In this scenario, different activities are performed that involve monitoring indoor patients and hospital staff. In addition, the movements of rescue team members are also monitored. The network scenario is divided into three regions. Each region involves three events. All monitored events are reported to the control room. The obtained simulation results are quite convincing and nearly identical to realistic experimental results. The main goal of the simulation is to accelerate the disaster recovery process by consuming the less energy and improving the QoS provisioning. We deploy the PT model with O-MAC to evaluate the performance and compare it with the following hybrid protocols: the enhanced cross-layer protocol (ECLP) [29], cross-layer MAC (CL-MAC) [30], pipeline-featured MAC (P-MAC) [31], routing-enhanced-duty-cycle MAC (RMAC) [32], MAC cross-layer (MAC-CROSS) [33], the unified cross-layer protocol (XLM) [34], and the energy optimization protocol (EOA) [35]. Similar parameters have been used to simulate competing hybrid protocols. 

The network contains 450 sensor nodes that are randomly deployed over a 1200 × 1200 m^2^ field. The network is divided into three regions and each region is handled and controlled by a DON. We initially let the DON remain at a corner of each region and distribute the sensor nodes in the three regions. When the simulation begins, the mobile sensor nodes move back and forth in the network regions and initiate their assigned responsibilities. Each simulation run lasts for 45 min. Furthermore, five routing protocols are deployed: PT, the multi-speed routing protocol (MMSPEED) [36], multi-constrained QoS multi-path routing (MCMP) [37], the energy aware-routing protocol (EAP) [38], and sensor protocols for information via negotiation (SPIN) [39].

We use a sensor application module with a constant bit-rate source that maintains QoS requirements [40]. The simulation parameters are presented in Table 7. We obtained several results but use the following metrics to demonstrate the performance of the PT model and O-MAC with PT:

■ Predicted pheromone rates at each link with respect to the number of delivered packets by deploying MCMP, MMSPEED, SPIN, EAP and PT.
■ Throughputs of MCMP, MMSPEED, SPIN, EAP, and PT with respect to the different mobility rates.
■ The end-to-end delays of packets using MCMP, MMSPEED, SPIN, EAP and PT.
■ The successful packet delivery rates and energy utilization efficiencies using the O-MAC and hybrid protocols.
■ Throughput performance of the O-MAC and hybrid MAC protocols.
■ Latency of the O-MAC and hybrid MAC protocols.

**Table 7:** Simulation parameters and their corresponding values.

Parameter	Value
Size of the WSN	1200 m × 1200 m
Size of each region	400 m × 400 m
Number of nodes	450
Routing protocols	MCMP, MMSPEED, SPIN, EAP and PT
Medium access control protocols	X-MAC, WiseMAC, B-MAC, and IEEE 802.15.4
Transmission range	30
Size of the packets	256 Bytes
Hybrid MAC protocols	O-MAC, ECLP, CL-MAC, P-MAC, RMAC, MAC-CROSS, XLM and EOA
Time for topology change	1.5 s
Propagation model	Deterministic
Sensing range of the node	30 m
Initial energy of the node	4 Joules
Bandwidth of the node	50 kb/s
Simulation time	45 min
Average number of simulation runs	15

### 5.1. Predicted Pheromone Rate at Each Link

**Figure 7:**
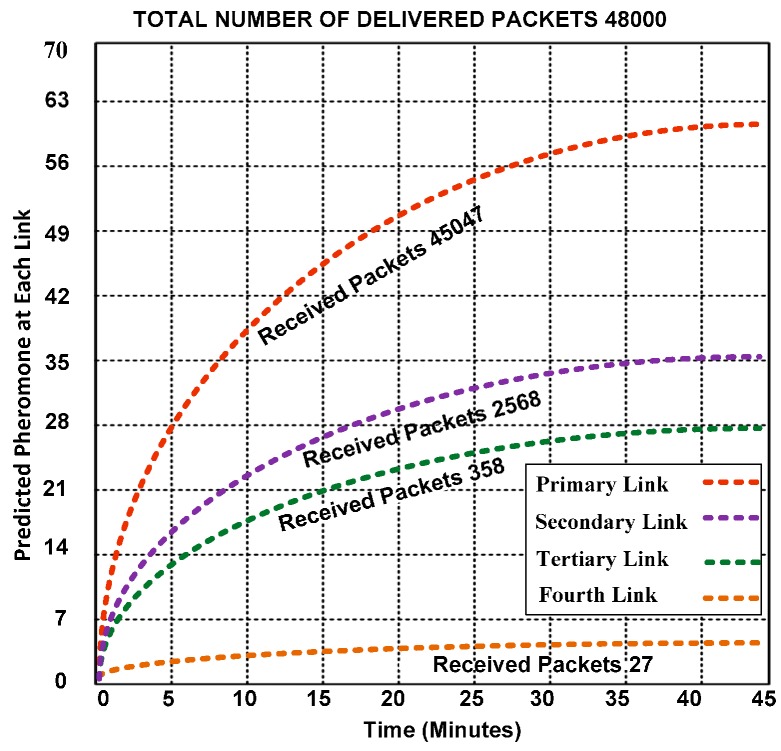
Predicted pheromone on multiple links.

We use four links to examine the trends of the arriving packets. The PT model always chooses the highest pheromone link to transfer data packets. Hence, it is important to choose suitable values for the pheromone generation “β” and pheromone sensitivity “P_s_”. Figure 7 indicates that the PT model can use secondary, tertiary, and other paths if the primary path is congested, which helps avoid congestion on the network and reduces the packet drop rate. The network transmits 48,015 data packets and only loses fifteen packets. Thus, approximately 99.97% of the packets are successfully delivered, which is a better outcome. 

The speeds of the sensor nodes vary from 0 to 18 m/s. The simulations demonstrate that O-MAC with PT produces a stable throughput, whereas MCMP, MMSPEED, SPIN, and EAP with O-MAC experience slight problems due to motion. As a result, the competing protocols have decreased throughputs. The simulation results demonstrate that PT with O-MAC is the superior choice for improving the QoS, whereas O-MAC-EAP, O-MAC-MCMP, O-MAC- MMSPEED and O-MAC-SPIN result in low throughput because of a lack of mobility features.

The primary link shares 93.85% of the successfully delivered packets. Choosing the primary path while delivering additional packets helps reduce the energy consumption because the specific nodes on the path can collaborate to send the packets while the other nodes are in the sleeping state. These results confirm that PT provides a higher throughput and reduced congestion. In the case of broken links, PT provides the flexibility to choose an alternate path that saves the network from congestion. As a result, the throughput remains stable.

### 5.2. Throughput of MCMP, MMSPEED, SPIN, EAP and PT

**Figure 8:**
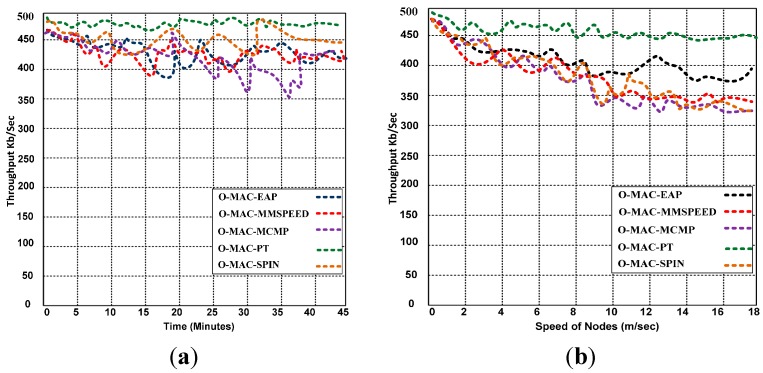
(**a**) Throughput when the nodes are all static and (**b**) Throughput when 50% of the nodes are mobile.

We evaluate the throughput efficiency of each routing protocol in Figure 8. PT appears to be compatible with O-MAC. In Figure 8, we present the results of simulations of mobile and static scenarios using MCMP, MMSPEED, SPIN, EAP, and PT with O-MAC. To examine the robustness of these five routing protocols, we simulate a combined mobile and static scenario. The static sensor nodes are fixed in the three monitoring regions of the network, whereas the mobile nodes are attached to people (e.g., rescue team members, doctors, nurses, and other staff) and the moving vehicles being used in the affected area to rescue the injured and victims. We analyze the flow of communication and delivered successful data (throughput) when performing the different activities in the three regions.

### 5.3. End-to-End Delay of Packets Using MCMP, MMSPEED, SPIN, EAP and PT

**Figure 9:**
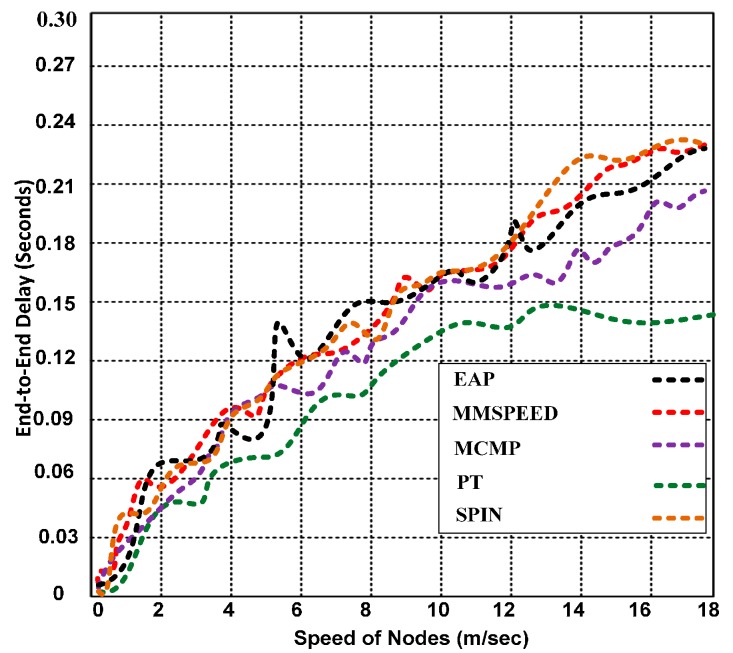
End-to-end delay for PT, SPIN, MCMP, MMSPEED and EAP.

The end-to-end delay is one of the most significant parameters for improving the QoS for WSNs. Many WSN applications require an end-to-end delay for time-sensitive data. However, this delay is difficult to bound for event-driven WSNs, in which the sensor nodes produce and broadcast data only when an event of interest occurs, thus creating a variable traffic load. The end-to-end delay is also tightly linked to other factors, such as the network capacity, energy, and the relative location of the sensors and sink nodes. In real-time applications, packets are dropped when they are transmitted with excessive delays. Figure 9 presents the end-to-end delays of PT, MCMP, MMSPEED, SPIN and EAP. 

PT produces a lower end-to-end delay than do the other competing protocols, and the end-to-end delay and node density are inversely proportional in the PT algorithm, which aids in finding alternative paths to the destination. PT also exhibits higher route maintenance than do SPIN, MCMP, MMSPEED, and EAP due to the efficient use of alternative paths, which helps produce low end-to-end delays even with mobile nodes. 

### 5.4. Successful Packet Delivery Rate and Energy Utilization Efficiency of O-MAC and Competing Hybrid Protocols

In this section, we analyze and evaluate the performance of the O-MAC and other competing hybrid (cross-layered) protocols: ECLP, CL-MAC, P-MAC, RMAC, MAC-CROSS, XLM and EOA. We created a mobility scenario with 80% of the nodes as mobile nodes and monitored the activities of all four regions. There is one problem noticed in the mobile scenario, which occurs when the nodes occasionally exceed the transmission range. Thus, the nodes require the dynamic sink (head node) to reduce its energy consumption; in our case, we use a DON to handle this issue. 

**Figure 10:**
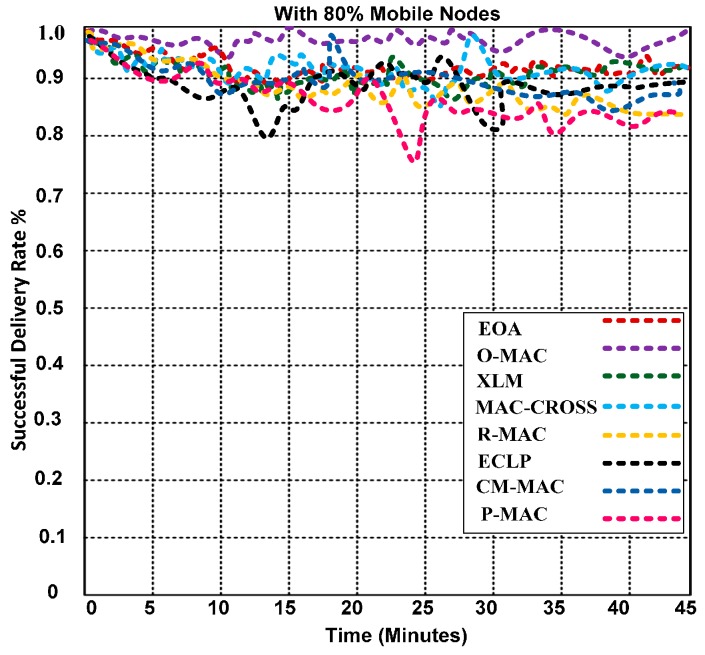
Successful packet delivery rates of O-MAC and other competing hybrid MAC protocols with 80% mobile nodes.

We used a node speed varying from 0 to 18 m/s. Figure 10 illustrates that O-MAC outperforms the other competing protocols in terms of the successful delivery of packets. The O-MAC protocol has a higher packet delivery rate because of its efficient use of several features, such as semi-synchronization, the anycast methodology, randomization, the ODFF, automatic buffering, and determination of a node’s information prior to leaving the communication; whereas the competing hybrid protocols lack these features.

**Figure 11:**
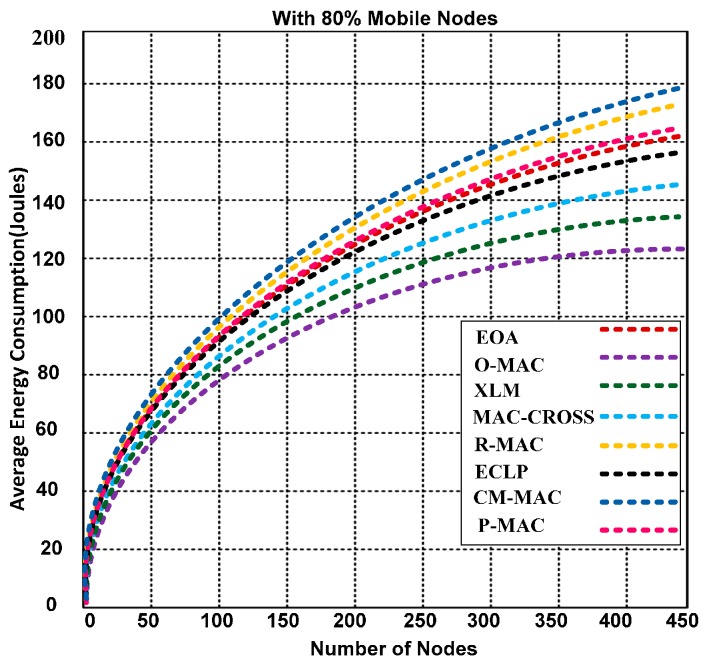
Energy consumption of O-MAC and other competing hybrid MAC protocols with 80% mobile nodes.

The performance of O-MAC and competing hybrid MAC protocols were analyzed in highly congested networks. O-MAC outperforms all competing hybrid protocols. The success rates of the competing protocols decrease when the simulation time increases. Figure 11 illustrates that O-MAC consumes less energy than the other competing MAC protocols. The reason for the lower energy consumption is the use of a short preamble without incorporating the address with the preamble. The short preamble reduces the time and energy consumption of each node in the network. In addition, O-MAC has the support of the PT model, which includes two important features: the packet generation rate and pheromone sensitivity to help find a reliable and robust path for forwarding the packets over single and multiple links, which leads to lower energy consumption and increased efficiency.

### 5.5. Throughput Performance of O-MAC and Other Hybrid MAC Protocols

**Figure 12:**
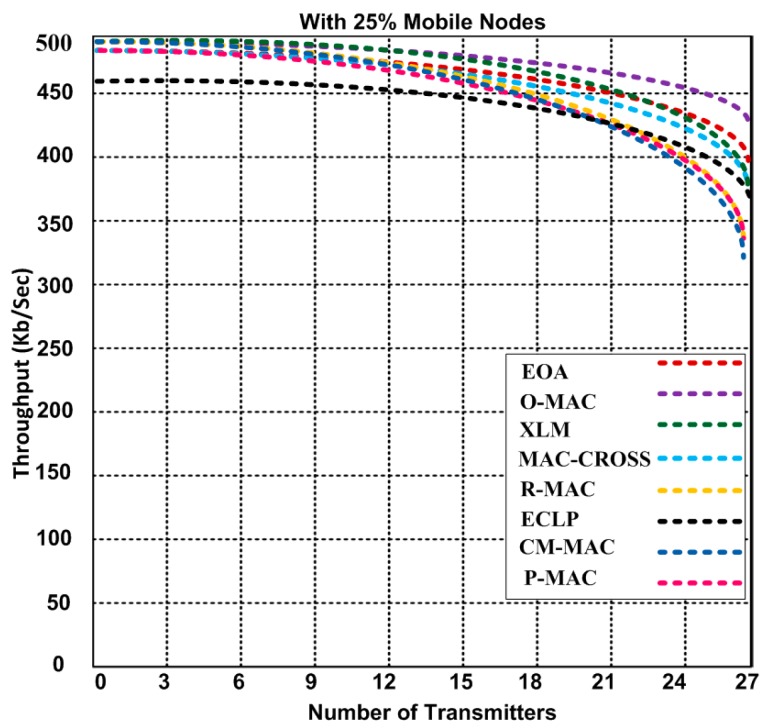
Throughput of O-MAC and other competing hybrid MAC protocols with a varying number of transmitters.

We analyze the throughput efficiency of O-MAC and other competing hybrid MAC protocols. We used combined static and mobile scenarios for determining the throughput based on the varying number of transmitting nodes. In Figure 12, we set 25% of the nodes to be mobile, including transmitting nodes throughout the simulation. O-MAC and other competing MAC protocols initially produce an average throughput of 458 to 500 kb/s, but when the number of transmitting nodes increases, the performance of O-MAC decreases only slightly compared with other hybrid MAC protocols. The O-MAC throughput decreases from 500 to 439 kb/s, whereas the throughput of the other hybrid MAC protocols decreases from 458 to 324 kb/s with the same number of transmitters. O-MAC is superior to the other competing MAC protocols and achieves a throughput that is 8.4% to 23% higher. This mobility analysis is measured using two methodologies: analysis based on synthetic traces and analysis based on real-world traces, as discussed in [41].

### 5.6. Latency

In this section, we introduce the latency by using O-MAC and other competing hybrid MAC protocols. We measure the latency in terms of how much time one packet takes to travel from the sender to the destination point. 

**Figure 13:**
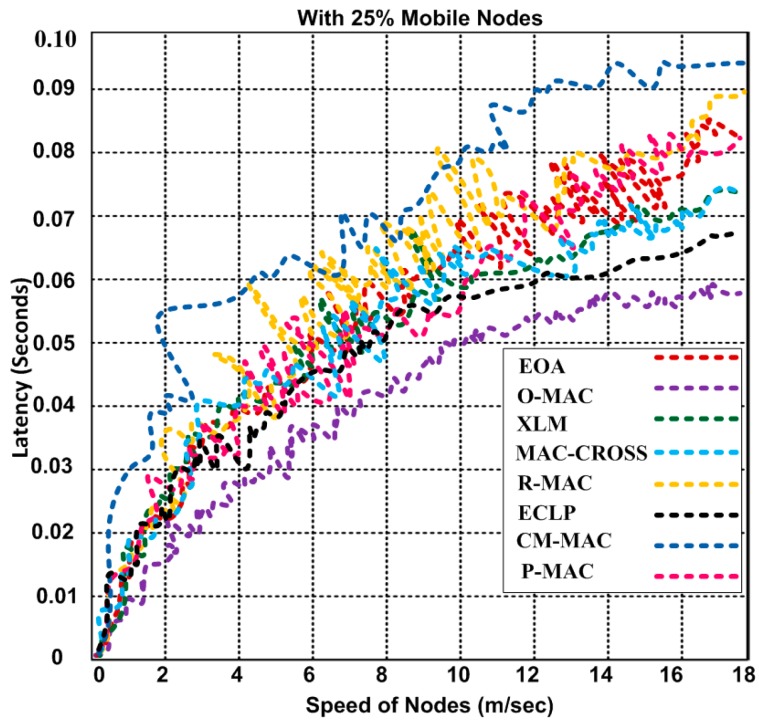
Latency of O-MAC and other competing hybrid MAC protocols with varying mobile speeds.

In addition, we measure and display different types of latencies, including the propagation delay, transmission delay, router delay and storage delay. These four types of delays are collectively shown in Figure 13. We also used the combined mobile and static scenario for determining the latency.

The latency at the different speeds is shown in Figure 13. The simulation illustrates that the latency increases with increasing mobility. O-MAC has 0.0012–0.058 s of latency at speeds of 0–18 m/s with 25% mobility, whereas other competing MAC protocols show higher latency, namely, 0.0013–0.094 s for the same number of mobile nodes. CM-MAC produces higher latency than the other hybrid MAC protocols. O-MAC achieves 9.23%–37.07% lower latency than the other MAC protocols. We validate that O-MAC can be used for different types of applications that require a faster delivery of data. Based on the simulation, our modular energy efficient paradigms based on MAC and network layers substantially improved the energy efficiency and QoS provisioning. The improvement of our proposed paradigms is shown in Table 8.

**Table 8:** The improvement of the proposed O-MAC with the PT model compared with other competing hybrid protocols.

Metric	Improvement in Percentage
Energy consumed in accessing the channel	5.4% to 8.2%
Energy consumed in sending the short preamble	13.5% to 18.4%
Energy consumed in forwarding 5 MB of data	3% to 4.2%
Energy efficiency	3.88% to 31.84%
Successful packet delivery rate	8.2% to 15.4%
Throughput with a varying number of transmitters	8.4% to 23%
Latency	9.23% to 37.07%
Channel access time	18.9%
Data frame transfer time	2.3%
Retry transmission time	27.3%
Retry transmission time	11.1%
Total channel access, frame transfer and acknowledgment and turnaround time in an ideal scenario	8.9%
Total channel access, frame transfer and acknowledgment and turnaround time in the worst-case scenario	8.7%

## 6. Conclusions

This paper presents modular energy-efficient paradigms that comprise two archetypes: the operational medium access control protocol and pheromone termite model. The O-MAC reduced the overhearing and congestion and improved the energy efficiency by deploying semi-synchronization, the anycast methodology, randomization, dynamic node selection and determination of the node’s information prior to leaving the communication. O-MAC also involves an ODFF model for the carrier sense multiple access portion. This model reduces the channel access time, data forwarding time, acknowledgement time, and retry transmission time. As a result, there is a substantial reduction in the energy consumption and improvement of the QoS parameters. The PT paradigm provided robust and reliable routing for single and multiple paths in disaster recovery scenarios. Two important features of the PT model are introduced: the packet generation rate and pheromone sensitivity. These features helped avoid congestion and improve the QoS. The performance of O-MAC with the PT model is a tradeoff between energy efficiency and QoS provisioning. Thus, several metrics were investigated and demonstrated the improvement achieved by our proposed paradigms. 

To investigate the effectiveness of paradigms, the ns3.10 network simulator with Ubuntu 14.04 was used to demonstrate the strengths of O-MAC with the PT model in a realistic disaster recovery scenario. O-MAC with PT improved the throughput and reduced the latency and energy consumption in the mobile and static scenarios. Furthermore, we compared O-MAC with known low-duty-cycle protocols (X-MAC, LPL and IEEE 802.15.4) and found improvements in terms of time savings and energy efficiency. These results demonstrate that O-MAC with PT could be used for several application areas over large-scale WSNs. These paradigms have marginal limitations at the transport level. Thus, in the future, we will lessen these limitations by introducing transport-level synchronization to re-order the packets, guaranteeing consistent pre-packet delivery for different packet types and faster recovery in the case of packet loss.
